# Persistent establishment of a tropical disease in Europe: the preadaptation of schistosomes to overwinter

**DOI:** 10.1186/s13071-019-3635-0

**Published:** 2019-07-29

**Authors:** Stephen Mulero, Olivier Rey, Nathalie Arancibia, Santiago Mas-Coma, Jérôme Boissier

**Affiliations:** 1 IHPE, University of Montpellier, CNRS, Ifremer, University of Perpignan Via Domitia, 66860 Perpignan, France; 2 Departamento de Parasitología, Facultad de Farmacia, Universidad de Valencia, Av. Vicent Andrés Estellés s/n, Burjassot, 46100 Valencia, Spain

**Keywords:** Schistosomiasis, *Bulinus truncatus*, Corsica, Persistence, *Schistosoma haematobium*, Temperature

## Abstract

**Background:**

Global changes promote the spread of infectious diseases worldwide. In this context, tropical urogenital schistosomiasis is now permanently established in Corsica since its first emergence in 2013. The local persistence of the tropical pathogens (schistosomes) responsible for urogenital schistosomiasis at such latitudes might be explained by (i) the presence of its intermediate host, the snail *Bulinus truncatus*, (ii) the recurrent local reseeding of schistosomes by their vertebrate hosts (either human or animal) every summer, and/or (iii) the maintenance and survival of schistosomes within their snail hosts over winter.

**Methods:**

In this study we conducted an ecological experiment to assess the ability of temperate and tropical schistosome strains to survive in classical winter temperatures in Corsican rivers when infecting temperate (local) snail strains. We also quantified the ability of the schistosomes to complete their life-cycle post-overwintering when returned to classical summer water temperatures.

**Results:**

Our results show that Mediterranean molluscs are locally adapted to winter conditions compared to tropical molluscs. Moreover, temperate and tropical schistosome strains equally survived the cold and produced viable offspring when returned to optimal temperatures. These results indicate that schistosomes can overwinter under temperate climates when infecting locally adapted snails and might partly explain the establishment and maintenance of schistosomes in Corsica from year to year.

**Conclusions:**

The observed broader thermal range of schistosomes compared to that of their snail hosts was unexpected and clearly indicates that the spread and establishment of schistosomiasis in temperate countries relies primarily on the presence of the locally adapted snail host lineages, currently known to be present in France, Italy, Portugal, Spain and Greece.

## Background

There is an increasing interest in the effects of global changes on the emergence and spread of infectious diseases worldwide. Both environmental and anthropogenic changes are expected to promote the geographical spread of pathogens. In particular, tropical infectious diseases are expected to migrate towards more temperate regions [1]. Empirical cases of tropical infectious disease emergence in temperate areas are still scarce with most reports being associated with diseases transmitted by arthropod vectors (e.g. dengue, malaria and chikungunya) [2–5]. Considering the biology of snail vectors (in particular their limited dispersal ability), the emergence of tropical snail-borne diseases was not expected in Europe. However, clusters of autochthonous urogenital schistosomiasis cases were identified in southern Europe in 2014. Urogenital schistosomiasis is caused by the trematode species *Schistosoma haematobium*, a parasite transmitted to humans by freshwater *Bulinus* snails [6]. This disease is one form of bilharziasis, a disease including a range of diseases responsible for more than 240 million cases in 78 countries across the world and responsible for between 1.7 and 4.5 million disability-adjusted life years (DALYs) every year [7, 8].

In April 2014, cases of urogenital schistosomiasis were simultaneously diagnosed in French and German hospitals with several local people infected [9–11]. Surprisingly, these European patients had never visited schistosomiasis endemic areas before (i.e. Arabian Peninsula or Africa [12]) and all patients were infected in August 2013 in Corsica, a French Mediterranean Island very popular for its touristic attractiveness [9–11]. The population in Corsica jumps from 300,000 people during the winter to 3 million people during the summer season. After a huge diagnostic campaign including 30,000 participants, 106 cases of urogenital schistosomiasis were linked to the water contact, by the patients, at specific sites on the Cavu River in southern Corsica. According to an epidemiological model built on an exhaustive dataset from the summer 2013 outbreak at the country scale, up to 338 (95% confidence interval 166–510) people could have been infected in Corsica, several of whom are probably asymptomatic with low level infections that will not be diagnosed.

In the summer of 2014, human access to the Cavu River was prohibited. However, in the summer of 2015 and 2016 new cases of local infections were identified hence strongly suggesting that transmission was ongoing and established in Corsica, with the life-cycle persisting in the local snails and tourists/locals [14, 15]. Moreover, genetic and phylogenetic analyses indicated that the schistosome strain established in Corsica is in fact a hybrid lineage resulting from the interbreeding between *S. haematobium* and *S. bovis* (a ruminant-associated schistosome species) highly likely originating from northern Senegal [16, 17].

In parallel with human diagnostics and surveillance, the snail intermediate hosts have regularly been collected and screened for possible schistosome infections every summer since 2014 [15]. *Bulinus* snails were collected weekly at the identified transmission sites along the Cavu River during the touristic season (from mid-June to mid-September), which also corresponds to the optimal period for transmission [17]. In particular, over this period, the water temperature is optimal for the development and release of schistosome cercarial larval stage from the infected snails, and the densities of potentially infected snails and humans are conjointly maximal. In 2014 and 2015, 3544 and 1965 snails, respectively, were tested for patent schistosome infections by cercarial shedding (i.e. looking for the emergence of schistosome cercarial larvae) and then in 2016 and 2017, 3453 and 5364 snails, respectively, were screened for schistosome infection using a more sensitive PCR diagnostic assay. Surprisingly, from 14,326 snails analysed no positive snails were detected despite cases of urogenital schistosomiasis being diagnosed and hence confirming ongoing transmission [15]. This suggests that although there is little doubt that the schistosome persists at some specific sites, the schistosome density is locally low.

How the parasite maintains itself at such latitudes and, in particular, how parasites cope with local winter conditions still remain enigmatic [12]. Two non-exclusive hypotheses might explain the overwintering persistence of the parasite. First, only adult parasites in their human hosts survive during the winter and transmission persists through yearly release of schistosome eggs from infected individuals. Under this hypothesis, the persistence of the parasite year to year only relies on the release and re-seeding of schistosomes in the river every early summer by humans [13]. Secondly, at least some schistosome larvae survive during winter within their snail hosts. Under this hypothesis, infected snails, even if they are scarce, could contribute to the infection of humans from one summer season to another. This hypothesis implies that (i) infected local snails survive at low water temperatures over the winter, and (ii) schistosomes within their snail hosts also survive during the winter and can develop into mature cercariae to complete their life-cycle after wintering. However, the observed longevity of infected adult snails under classical experimental rearing conditions (i.e. temperature around 25 °C, photoperiod 12:12 h L:D) is generally low. Moreover, the environmental conditions during winter in temperate regions appear at first sight unfavourable for tropical parasites with complex life-cycles. As a result, this latter hypothesis is generally discarded.

In this study we experimentally test this last hypothesis. To this aim, we conducted a mesocosm experiment to (i) quantify the survival of healthy and infected snails and that of their schistosomes within their hosts to an experimental yet realistic temperate winter cycle; and (ii) quantify the ability of the schistosomes to complete their life-cycle after wintering. We measured the life history traits of different *Bulinus*/*Schistosoma* strain couples, including *Bulinus* from different tropical and temperate countries to test for potential local adaptation in temperate regions, and tropical schistosomes either pure *S. haematobium* or hybrids, to test for possible schistosome strain effects on both the snail and the schistosome survival in relation to cold stress.

## Methods

### Snail and schistosome strains

Three *Bulinus truncatus* (*B.t*) strains originating from Cameroon, Spain and Corsica and two *S. haematobium* (*S.h*) strains originating from Cameroon (pure *S.h*) and Corsica (*S. haematobium-bovis* hybrid (*S.h-b*) were used in this study.

The *B.t* strain from Cameroon originated from the Barombi Kotto Lake (4°28′04″N, 9°15′02″W) in the southeast of Cameroon and was isolated in July 2015; the *B.t* strain from Spain originated from Almeria (36°44′54.8531″N, 2°47′54.0978″W) and was isolated in March 2015 and the *B.t* strain from Corsica was obtained from the Cavu River (41°43′26.86″N, 9°17′55.09″E) and was isolated in April 2014. All of these snail strains were reared under common laboratory environmental conditions for at least two generations before starting the experiment. This period was applied to avoid possible environmental and maternal effects on the observed response to the subsequent cold stress experiment.

The *S.h* strain from Cameroon originated from Barombi Kotto (4°28′04″N, 9°15′02″W) and was isolated in July 2015 from naturally-infected children [18] and the Corsica *S.h-b* hybrid strain was isolated from patients infected in Corsica in the summer of 2013 [17]. Nuclear (ITS2) and mitochondrial (*cox*1) gene sequencing revealed that the Cameroon *S.h* is a pure *S. haematobium* strain [18] and Corsica strain *S.h-b* is a hybrid strain resulting from the interbreeding between *S. bovis* and *S. haematobium* most likely originating from Senegal [17]. These two schistosome strains have been routinely maintained in the laboratory facilities using hamsters (*Mesocricetus auratus*) as definitive hosts and the strain-specific *B. truncatus* as the intermediate host (*B.t* Corsica for *S.h-b* Corsica and *B.t* Cameroon for *S.h* Cameroon) following classical protocols developed by our laboratory [19, 20].

### Snail infection

Schistosome eggs from the two strains were isolated from the liver of independently infected hamsters and then placed in freshwater and exposed to light to induce miracidial hatching. A total of 8 batches of size-normalised snails each (i.e. 3–5 mm) were individually exposed to 5 schistosome miracidia to enable infection. These 8 batches included 4 batches of Corsican *B.t* exposed to the *S.h*-*b* from Corsica and 4 batches of Spanish *B.t* exposed to *S.h* from Cameroon. After exposure, snails were maintained in batches of 50 individuals in 5-l containers at 24 °C for 6 weeks. After 6 weeks, the exposed snails were individually placed in fresh water in a well of a 24-well plate and exposed to light for 2 h to stimulate cercarial shedding. Shedding snails were observed under a binocular microscope and only snails emitting cercariae were kept. From these infected snails we constituted 8 batches of 12 individuals maintained in 500 ml tanks. These 8 batches correspond to the eight snail/schistosome combinations tested (Table 1). Twelve other batches of 12 non-exposed snails were constituted and maintain in 500 ml tanks. These 12 batches included 3 batches of each snail strain (Cameroon, Corsica and Spain).

**Table 1:** Mollusc and parasite combinations tested during the winter experiment

Parasite/mollusc	*S. haematobium* Corsica (*S.h-b*)	*S. haematobium* Cameroon (*S.h*)	Uninfected
*B. truncatus* Corsica (*B.t* Corsica)	(12)*4	Not tested	(12)*4
*B. truncatus* Spain (*B.t* Spain)	Not tested	(12)*4	(12)*4
*B. truncatus* Cameroon (*B.t* Cameroon)	Not tested	Not tested	(12)*4
Temperature treatments (°C)	24, 16, 8, 4	24, 16, 8, 4	24, 16, 8, 4

*Notes*: The values displayed represent the number of molluscs used for each combination. All of these combinations were replicated for each temperature treatments, representing 5 batches of 12 molluscs for each temperature and a total of 20 batches of 12 molluscs. Other combinations like *B.t* Cameroon and *S.h* Cameroon were not tested as they are not relevant to the present study (i.e. the establishment of tropical parasites in European *B. truncatus* molluscs)

### Temperature testing

**Fig. 1:**
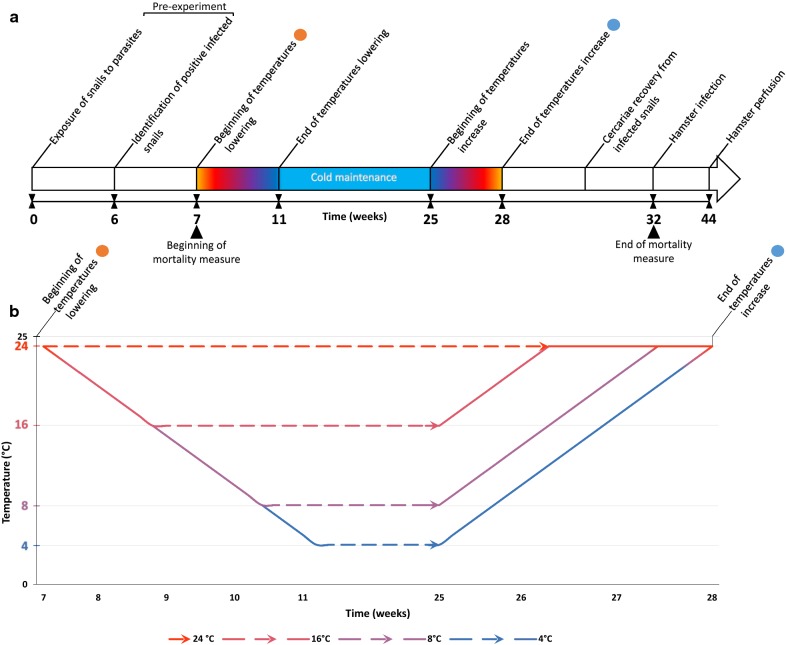
Time schedule of the experimental protocol. **a** The timing of the entire experiment. **b** The temperature kinetics of the winter experiment. All the values in abscissa represent the time in weeks between each step

**Fig. 2:**
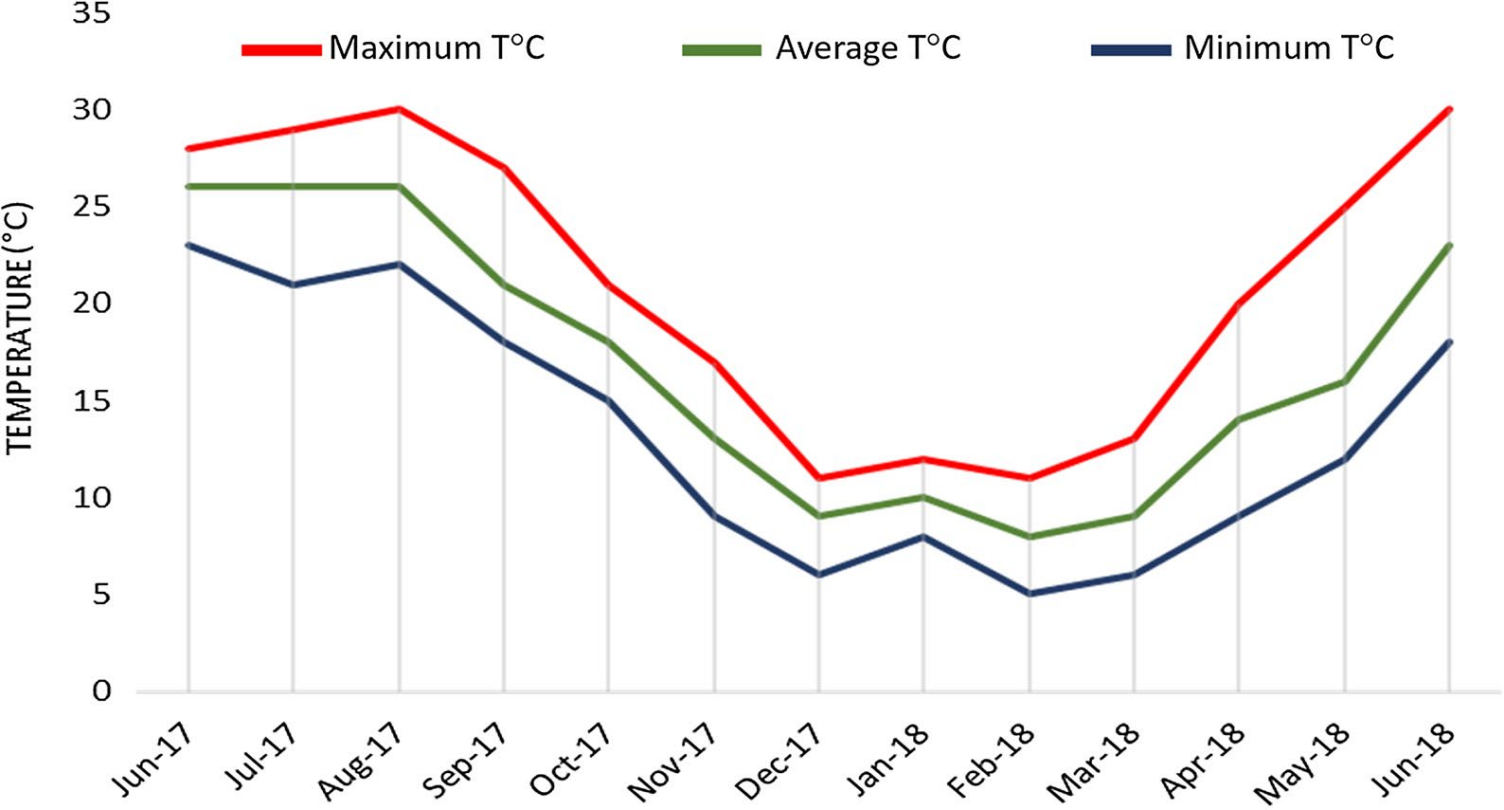
Temperatures recorded in the Cavu River from June 2017 to May 2018. Three sites where cases of urogenital schistosomiasis have been previously identified in the Cavu River: site 5, 41°42′16.82″N, 9°20′5.23″E; site 8, 41°43′12.22″N, 9°18′6.27″E; and site 9, 41°43′53.14″N, 9°17′36.87″E. The values recorded for each of the three sites are pooled and averaged over the month

The temperature testing consisted of a four-step protocol (Fig. 1): a progressive decrease in temperature over 4 weeks; a long-term maintenance at specific “winter temperature” for 14 weeks; and a progressive increase in temperature over 3 weeks with cercarial shedding checked for a further 4 weeks. One control temperature (24 °C) and three “winter temperatures” (4, 8 and 16 °C) were tested for each infection (Table 1). This range of temperatures was selected to mimic the thermal dynamics measured *in natura* at three sites where the transmission of urogenital schistosomiasis was previously identified in the Cavu River [17] (Fig. 2). A temperature of 24 °C is optimal for snail and schistosome development leading to cercarial shedding and was used as a control. This optimal temperature also corresponds to normal summer temperature experienced by natural snail populations in the Cavu River (Fig. 2).

The study was conducted by placing the different tanks of 500 ml containing the snails in different 60-l freshwater tanks maintained at the different required temperatures (bain-marie). For the uninfected and infected control batches, kept at the normal temperature of 24 °C, the 500-ml freshwater containers were maintained on a laboratory bench in the snail room which is routinely kept at 24 °C. After acclimatisation for one week at 24 °C, the temperature was lowered at a rate of 1 °C every 2 days until the experimental winter temperatures reached 16 °C, 8 °C and 4 °C, respectively. The temperature was lowered using chiller devices (AquaVie Ice 1200/2000; Aqua Store, Dagneux, France) and a water pump system to mix the water to maintain a constant temperature throughout the bain-marie. For the batches maintained at 4 °C, the containers were placed in a fridge at 4 °C. All batches of snails, independent of temperature, were exposed to the same photoperiod (12:12 h L:D). A total of 4 weeks was thus needed to progressively decrease the temperature for the different batches after which they were maintained for 14 weeks for the 8 °C and 16 °C batch and 10 weeks for the 4 °C batch. After this cold exposure, the temperature was progressively increased at a rate of 1 °C/day for all aquaria until the control temperature was reached (i.e. 24 °C).

### Checking for schistosome survival after the cold exposure

Once the snails had been returned to their normal temperature of 24 °C they were individually checked for cercarial shedding as described previously. This was done on a weekly basis for 4 weeks. Pooled cercariae coming from the *S.h* Corsican strain maintained at 8 °C were used to expose hamsters. A total of 400 cercariae were used to individually expose 3 hamsters using the routine procedure [19, 21, 22]. Hamsters were sacrificed 90 days after cercarial exposition and adult schistosomes were recovered using the hepatic perfusion technique [19, 21, 22].

During all the experiments, snails were fed *ad libitum* with fresh lettuce 3 times a week. All juvenile snails that hatched from eggs laid during the experiment were removed. Snail mortality was monitored once a week, from the beginning of temperature lowering period until the end of the shedding inspection period (i.e. a total of 25 weeks). Individual mortality was visually inferred by inspecting the presence/absence of heartbeats (through the shell transparency) under a binocular microscope. This trait is commonly measured to infer the effect of schistosomes on the snail health [23]. All confirmed dead individuals were recorded and removed from the aquariums.

### Data analysis

Mortality data were used to generate survival tables and Kaplan-Meier curves. Snail survival distributions were compared using log-rank tests in SPSS v.23.0 (IBM, New York, USA).

## Results

All data from the different batches of snails are shown in Table 2.

**Table 2:** Survival for each mollusc-parasite combination observed at the end of the experiment

Healthy molluscs		Infected molluscs	
T (°C): Mollusc strain	Survival (%)	T (°C) Mollusc/parasite strain	Survival (%)
4 °C: *B.t* Spain	58 (*n* = 7)	4 °C: *B.t* Spain/*S.h*	33 (*n* = 4)
8 °C: *B.t* Spain	92 (*n* = 11)	8 °C: *B.t* Spain/*S.h*	83 (*n* = 10)
16 °C: *B.t* Spain	75 (*n* = 9)	16 °C: *B.t* Spain/*S.h*	100 (*n* = 12)
24 °C: *B.t* Spain	83 (*n* = 10)	24 °C: *B.t* Spain/*S.h*	17 (*n* = 2)
4 °C: *B.t* Corsica	50 (*n* = 6)	4 °C: *B.t* Corsica/*S.h-b*	42 (*n* = 5)
8 °C: *B.t* Corsica	92 (*n* = 11)	8 °C: *B.t* Corsica/*S.h-b*	83 (*n* = 10)
16 °C: *B.t* Corsica	83 (*n* = 10)	16 °C: *B.t* Corsica/*S.h-b*	83 (*n* = 10)
24 °C: *B.t* Corsica	92 (*n* = 11)	24 °C: *B.t* Corsica/*S.h-b*	8 (*n* = 1)
4 °C: *B.t* Cameroon	0 (*n* = 0)		
8 °C: *B.t* Cameroon	0 (*n* = 0)		
16 °C: *B.t* Cameroon	100 (*n* = 12)		
24 °C: *B.t* Cameroon	100 (*n* = 12)		

*Notes*: Number of molluscs in parentheses (*n*). All infected molluscs emitted cercariae at the end of the experiment

*Abbreviations*: *B.t*, *Bulinus truncatus*; *S.h*, *Schistosoma haematobium* pure strain from Cameroon; *S.h-b*, *Schistosoma haematobium* × *S. bovis* Corsican hybrid strain; T, temperature

### Survival of uninfected snails

**Fig. 3:**
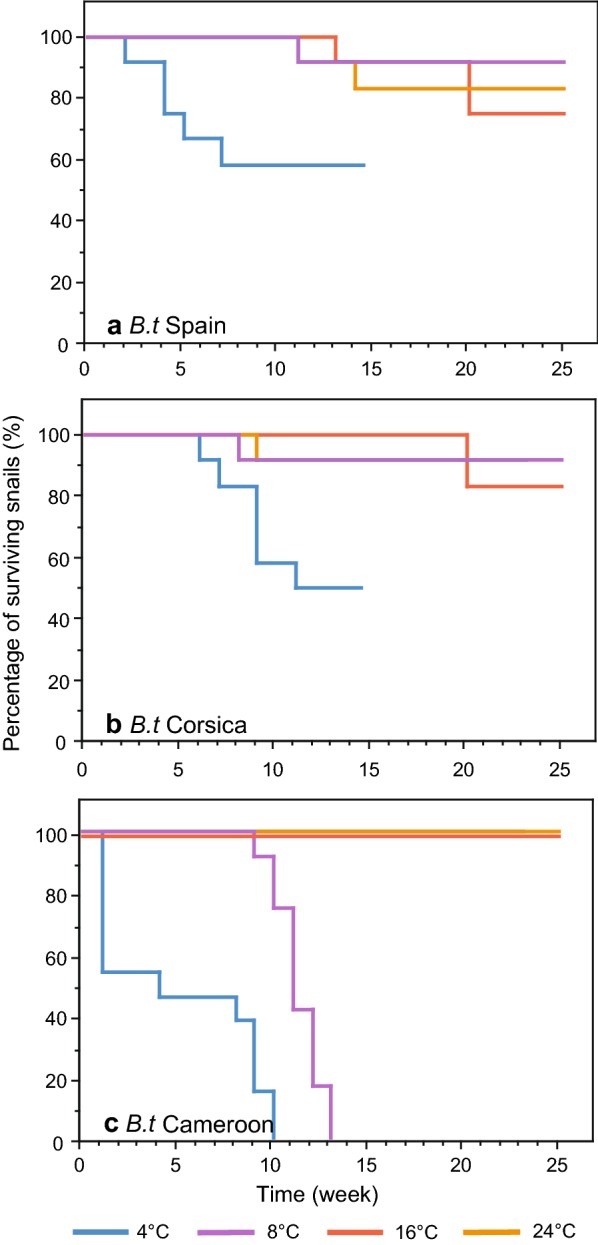
Survival curves of the healthy *B. truncatus* snails from the two temperate strains, Spain (**a**) and Corsica (**b**), and from the tropical strain, Cameroon (**c**), maintained at four different winter temperatures. *Key*: 4 °C, yellow curve; 8 °C, orange curve; 16 °C, blue curve; 24 °C, green curve

Survival curves for the three strains of uninfected *B.t* strains are presented in Fig. 3. No mortality was observed during the temperature lowering period. The survival curves obtained for the snails maintained at the four experimental temperatures were significantly different irrespective to the snail strain (Corsica: *χ*^2^ = 13.79, *df* = 3, *P* < 0.01; Spain: *χ*^2^ = 8.09, *df* = 3, *P* < 0.05; Cameroon: *χ*^2^ = 80.18, *df* = 3, *P* < 0.01). At 16 °C and 24 °C all *B.t* strains had a highest survival rate and we did not observe statistical difference between the three *B.t* strains (*χ*^2^ = 3.23, *df* = 2, *P *> 0.05 for 16 °C and *χ*^2^ = 2.03, *df* = 2, *P *> 0.05 for 24 °C). Conversely, significant differences were observed between the survival curves obtained from the *B.t* snails from Cameroon (tropical) and those from Corsica and Spain (temperate) when exposed to the two lowest temperatures (4 °C: *χ*^2^ = 14.47, *df* = 2, *P* < 0.01; 8 °C: *χ*^2^ = 31.55, *df* = 2, *P* < 0.01). In particular, all *B.t* from Cameroon died 10 weeks (4 °C) and 13 weeks (8 °C) after the beginning of the experiment while 50% of snails from Spain and Corsica survived when kept at 4 °C and 90% of the snails survived kept at 8 °C. No significant difference was observed between the survival curves obtained for the *B.t* from Spain and Corsica, irrespective to the temperature treatment (4 °C: *χ*^2^ = 0.007, *df* = 1, *P *> 0.05; 8 °C: *χ*^2^ = 0.001, *df* = 1, *P *> 0.05; 16 °C: *χ*^2^ = 0.31, *df* = 1, *P *> 0.05; 24 °C: *χ*^2^ = 0.305, *df* = 1, *P *> 0.05).

### Survival of infected snails

**Fig. 4:**
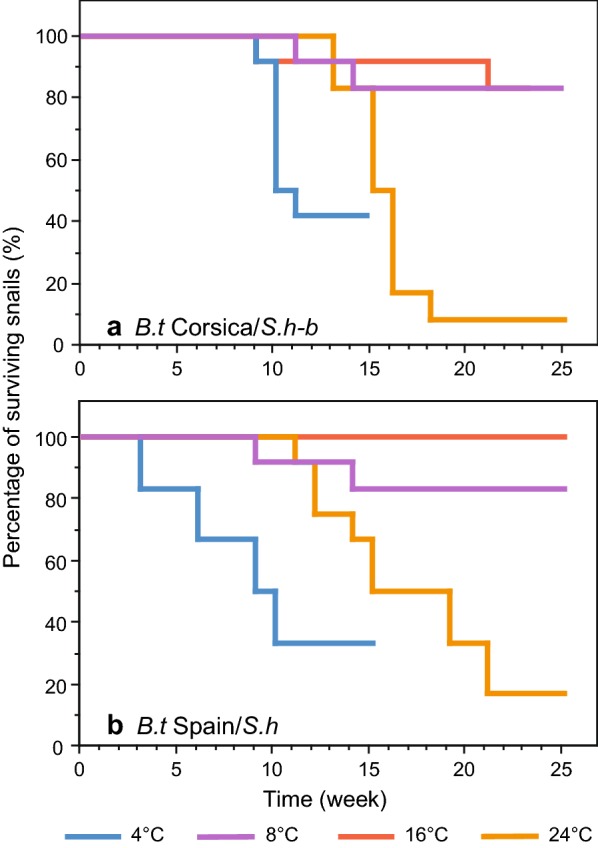
Survival curves of B. truncatus snails from temperate strains, Corsica (**a**) and Spain (**b**), infected with *S. haematobium* (*S.h-b*) from Corsica and from Cameroon (*S.h*) and maintained at four different temperatures. *Key*: 4 °C, yellow curve; 8 °C, orange curve; 16 °C, blue curve; 24 °C, green curve

The survival curves for the infected Corsican and Spanish *B.t* strains are shown in Fig. 4. No mortality was observed during the temperature lowering period. No significant differences were observed between the two host/schistosome combinations tested (4 °C: *χ*^2^ = 1.44, *df* = 1, *P *> 0.05; 8 °C: *χ*^2^ = 0.001, *df* = 1, *P *> 0.05; 16 °C: *χ*^2^ = 2.09, *df* = 1, *P *> 0.05; 24 °C: *χ*^2^= 0.60, *df* = 1, *P *> 0.05). The highest mortality was observed at 24 °C (the control temperature) with only 8 and 17% of Corsican and Spanish snails surviving, respectively. Moreover, no statistical difference was observed between the survival profile of the infected *versus* uninfected snails from either the *B.t* Corsica or the *B.t* Spain strains at 4 °C (*χ*^2^ = 0.37, *df* = 1, *P *> 0.05), 8 °C (*χ*^2^ = 0.66, *df* = 1, *P *> 0.05) or 16 °C (*χ*^2^ = 1.40, *df* = 1, *P *> 0.05).

### Cercariae shedding and infectivity

Four weeks after the snails had been returned to their natural temperature of 24 °C all the infected snails who survived the colder temperatures were still infected and emitted schistosome cercariae, independent of the temperatures that they had been maintained at. The number of surviving infected snails are presented in (Table 2). Pooled cercariae coming from the *S.h-b* Corsican strain maintained at 8 °C were used to expose hamsters. After 90 days of infection, 72, 84 and 104 adult worms successfully developed from each of the three exposed hamsters. These proportions are similar to the 25% cercariae infection success commonly obtained through routine laboratory passaging.

## Discussion

Our study demonstrates that (i) *B. truncatus* can survive during a long-term cold period; (ii) *B. truncatus* originating from temperate regions (i.e. Spain, Corsica) better survive to cold stress than the tropical strain (i.e. Cameroon); (iii) infected snails display a higher survival rate at cold temperature than at the theoretical (higher) optimal temperature, and (iv) the schistosomes inside their snail hosts survive cold stress for several weeks and produce viable cercariae once returned to optimal temperatures.

Our results show that *B. truncatus* is resilient to cold temperatures independent of its geographical origin and that snails from the temperate region (Corsica and Spain) resist better than snails from the tropical region (Cameroon). Even tropical snails from Cameroon can survive for periods at 16 °C for up to 25 weeks. This temperature is far lower than those experienced by the natural populations in Cameroon (i.e. higher than 29 °C) [24, 25] and experienced in most other tropical countries. Remarkably, *B. truncatus* strains from the temperate region (Spain and Corsica) are fully resistant to very low temperatures, such as 4 °C for at least ten weeks, which is lower than the temperatures observed in Cavu River during the winter. The strong difference in thermal resistance observed between temperate and tropical *B. truncatus* strains is a signature of local adaptation. This local thermal adaptation has been reported in several groups of plants or animals [26–29] but only in arthropods regarding disease vectors [30]. Local adaptations have been poorly studied in freshwater snails and studies have mainly focused on extreme environmental shifts, for examples the thermal adaptation of specific strains of *Radix balthica* (family Lymnaeidae) to some geothermal springs in northern Iceland has been reported [31].

The effect of temperature on the growth, fecundity and survival of their parasitic schistosomes within their snail hosts (*Bulinus* sp. and *Biomphalaria* sp.) has been recently reviewed [32]. This synthesis shows that, in the current context of global warming, most studies focused on the effect of increasing temperature with only a few case studies on the ability of snails to cope with colder conditions [32]. However, in light of the urogenital schistosomiasis outbreak in Corsica, it is also particularly relevant to quantify the effect of cold temperatures on snail life history traits to better predict the potential spread of *B. truncatus* and the risk of schistosomiasis transmission at higher latitudes. Similar cold resistance has been shown previously in uninfected snails from Egypt, although to less extreme temperatures, with only 2% and 6% of mortality after 2 weeks at 10 °C and 15 °C, respectively [33]. More recently, a study has investigated cold resistance of *B. globosus*, another schistosome snail vector in South Africa [34]. In this study, more than 85% of the snails survived after 12 weeks at 15.5 °C [34]. Together with our study, these findings indicate that *Bulinus* sp. can tolerate a range of temperatures including cold temperatures and are thus adapted to environments with fluctuating temperatures [35]; hence, *B. truncatus* snails are endemic in several temperate countries including France, Spain, Portugal, Greece and Italy [9, 12, 36, 37]. In comparison, *Biomphalaria* sp., a snail vector of human intestinal schistosomiasis displays a much narrower thermal niche [32] with *Biomphalaria tenagophila* being the only species of this genus that has been identified in a temperate climate (i.e. Romania [38]).

Our results also show that temperature has a significant impact on the survival of infected snails irrespective of the origin of the infecting schistosome (temperate or tropical region). Indeed, mortality substantially increased in infected snails kept at normal temperatures; however, it is interesting that snail survival rates were higher and similar in infected and uninfected snails at cold temperatures (4 °C, 8 °C or 16 °C). It is known that parasitism is expected to alter several fitness traits of their host snails, including thermal tolerance. We here interpret these results as an effect of cold stress on the development of schistosomes inside their host and not (or at least indirectly) on the snails. Indeed, in schistosome-snail interactions, temperature directly influences the development and several life history traits of the schistosome with optimal trait values observed around 25 °C [39]. Below this optimal temperature, the ability of miracidia to infect their host snails decreases [40–43], the length of the prepatent period increases [34, 41, 42, 44] and the number of cercariae emitted decreases [34, 41, 42, 44]. Our study shows that at 4 °C, 8 °C and 16 °C, the mortality of snails either infected or not does not differ significantly. This means that schistosome infection does not induce any fitness cost on snails additionally to cold stress. This might be explained by the fact that at these temperatures, the schistosomes enter diapause to overwinter and the possible effect of schistosomes on their hosts is thus limited. Conversely, unlike uninfected snails, whatever the strain considered almost all the infected snails died before the end of the experiment at 24 °C, as a consequence of a normal parasite development. In the light of these results we argue that cold temperatures inhibit the development and activity of the schistosomes within their hosts, hence maintaining the fitness of infected snails. In natural populations, infected snails are highly likely to overwinter in temperate countries as well as the uninfected populations. In relation to the Corsican outbreak it is highly possible that the maintenance of infected snails overwinter, at least partly, will contribute to the continued transmission of urogenital schistosomiasis from one summer to another. Importantly, however, schistosome infection intensity in snails is extremely low even during the summer [45] and so the contribution of overwintering infected snails in the maintenance of schistosomes in the river is likely to be very low.

Lastly, our results show that schistosomes better tolerate cold temperatures compared to their snail hosts, at least in the case of the tropical strains (i.e. from Cameroon). Indeed, all the uninfected *Bulinus* snails from Cameroon died after 13 weeks at 8 °C. Conversely schistosomes originating from all geographical areas, both temperate and tropical, can survive temperatures as low as 4 °C with the production of viable cercariae. Moreover, although not in all combinations of schistosome-snail strains, our results indicate that hybridisation between *S. haematobium* and *S. bovis*, which is the *S.h-b* Corsican strain (probably originating from Senegal) does not induce any changes in terms of thermal tolerance compared to the pure *S. haematobium* strain from Cameroon. Together these results strongly suggest that schistosomes from tropical areas, irrespective of their geographical origin and nature (i.e. pure or hybrid), are able to spread and settle in temperate regions, provided that locally adapted populations of *B. truncatus* are already established. In an epidemiological context, this means that the spread of schistosomiasis northward in Europe is currently limited by the adaptive potential of *B. truncatus* to colder temperatures and not by some physiological constraints of the schistosomes in relation to colder environments.

As a final point of interest, the overwintering period in temperate latitudes is analogous to the aestivation period known in tropical areas (see [46]). It is well established that several schistosome intermediate host species (including *B. truncatus*) have the ability to aestivate [46]. It has been demonstrated that aestivation negatively affects the survival of infected snails [47, 48]. In our experiments cold temperatures did not affect *B. truncatus* snails, regardless of their infection status. Interestingly, it has been evidenced that an aestivation period stops the sporocyst development [49] and does not affect cercariae [50]. Based on these previous studies and our results, we argue that the ability of schistosomes to enter dormancy is a pre-adaptation to cope with stressful environmental conditions experienced by their snail hosts.

## Conclusions

With ongoing climate change, temperature patterns can fluctuate drastically in time and space [51]. Here, we showed that temperatures can influence the biology of freshwater snails and the interactions with their schistosomes. The data support the hypothesis that the most limiting factor in assessing the ability of schistosomes to invade new areas is essentially limited by the physiological constraints of snail hosts to low temperatures. Thereby, the definition of the host’s thermal niche is a key element when estimating the risks of schistosomiasis spreading throughout southern Europe. Given the intensity of current temperature changes and their geographical variability, it is difficult to predict, on a large scale, the impact that this may have on the transmission dynamics of infectious diseases. These facts add a degree of complexity in the management of disease outbreaks [12]. For this purpose, ecological niche modelling approaches may be important for predicting the presence of *B. truncatus* snails in Europe and their ability to transmit medically important schistosome species. These tools were recently used to predict the distribution of snail species involved in schistosomiasis and fasciolosis transmission [52, 53] whilst also enabling predictions on the effect of climatic change and anthropogenic activities (e.g. land use), which also impact schistosomiasis transmission [54]. Previous studies have predicted a reduction of suitable ecological niches for three species of freshwater snails involved in parasitic disease transmission, and the appearance of suitable zones in areas that were previously unfavourable [52, 53]. These changes not only change the distribution of the snail hosts but also the parasites that they transmit.

## Data Availability

The dataset supporting the conclusions of this article is included within the article.

## Abbreviations

*S.h*: *Schistosoma haematobium* (pure strain)
*S.b*: *Schistosoma bovis* (pure strain)
*S.h-b*: *Schistosoma haematobium*-*bovis* (hybrid Corsican strain)
*B.t*: *Bulinus truncatus*
DALYs: disability-adjusted life years

## References

[1] Kim KH, Kabir E, Ara Jahan S (2014). A review of the consequences of global climate change on human health. J Environ Sci Health C Environ Carcinog Ecotoxicol Rev..

[2] Schaffner F, Fontenille D, Mathis A (2014). Autochthonous dengue emphasises the threat of arbovirosis in Europe. Lancet Infect Dis..

[3] Schaffner F, Mathis A (2014). Dengue and dengue vectors in the WHO European region: past, present, and scenarios for the future. Lancet Infect Dis..

[4] Gould EA, Gallian P, De Lamballerie X, Charrel RN (2010). First cases of autochthonous dengue fever and chikungunya fever in France: from bad dream to reality!. Clin Microbiol Infect..

[5] Rezza G, Nicoletti L, Angelini R, Romi R, Finarelli AC, Panning M (2007). Infection with chikungunya virus in Italy: an outbreak in a temperate region. Lancet..

[6] Rollinson D, Stothard JR, Southgate VR (2003). Interactions between intermediate snail hosts of the genus *Bulinus* and schistosomes of the *Schistosoma haematobium* group. Parasitology..

[7] WHO (2017). Schistosomiasis and soil-transmitted helminthiases: number of people treated in 2016. Wkly Epidemiol Rec..

[8] Hay SI, Abajobir AA, Abate KH, Abbafati C, Abbas KM, Abd-Allah F (2017). Global, regional, and national disability-adjusted life-years (DALYs) for 333 diseases and injuries and healthy life expectancy (HALE) for 195 countries and territories, 1990–2016: a systematic analysis for the Global Burden of Disease Study 2016. Lancet..

[9] Boissier J, Mone H, Mitta G, Bargues MD, Molyneux D, Mas-Coma S (2015). Schistosomiasis reaches Europe. Lancet Infect Dis..

[10] Holtfreter MC, Mone H, Muller-Stover I, Mouahid G, Richter J (2014). *Schistosoma haematobium* infections acquired in Corsica, France, August 2013. Euro Surveill..

[11] Berry A, Mone H, Iriart X, Mouahid G, Aboo O, Boissier J (2014). Schistosomiasis haematobium, Corsica. France. Emerg Infect Dis..

[12] Kincaid-Smith J, Rey O, Toulza E, Berry A, Boissier J (2017). Emerging schistosomiasis in Europe: a need to quantify the risks. Trends Parasitol..

[13] Noel H, Ruello M, Maccary A, Pelat C, Sommen C, Boissier J (2017). Large outbreak of urogenital schistosomiasis acquired in southern Corsica, France: monitoring early signs of endemicization?. Clin Microbiol Infect..

[14] Berry A, Fillaux J, Martin-Blondel G, Boissier J, Iriart X, Marchou B (2016). Evidence for a permanent presence of schistosomiasis in Corsica, France, 2015. Euro Surveill..

[15] Ramalli L, Mulero S, Noel H, Chiappini JD, Vincent J, Barre-Cardi H (2018). Persistence of schistosomal transmission linked to the Cavu river in southern Corsica since 2013. Euro Surveill..

[16] Mone H, Holtfreter MC, Allienne JF, Mintsa-Nguema R, Ibikounle M, Boissier J (2015). Introgressive hybridizations of *Schistosoma haematobium* by *Schistosoma bovis* at the origin of the first case report of schistosomiasis in Corsica (France, Europe). Parasitol Res..

[17] Boissier J, Grech-Angelini S, Webster BL, Allienne JF, Huyse T, Mas-Coma S (2016). Outbreak of urogenital schistosomiasis in Corsica (France): an epidemiological case study. Lancet Infect Dis..

[18] Kincaid-Smith J, Boissier J, Allienne JF, Oleaga A, Djuikwo-Teukeng F, Toulza E (2016). A genome wide comparison to identify markers to differentiate the sex of larval stages of *Schistosoma haematobium*, *Schistosoma bovis* and their respective hybrids. PLoS Negl Trop Dis..

[19] Boissier J, Chlichlia K, Digon Y, Ruppel A, Mone H (2003). Preliminary study on sex-related inflammatory reactions in mice infected with *Schistosoma mansoni*. Parasitol Res..

[20] Boissier J, Mone H (2001). Male-female larval interactions in *Schistosoma mansoni*-infected *Biomphalaria glabrata*. Int J Parasitol..

[21] Boissier J, Mone H (2000). Experimental observations on the sex ratio of adult *Schistosoma mansoni*, with comments on the natural male bias. Parasitology..

[22] Boissier J, Mone H (2001). Relationship between worm burden and male proportion in *Schistosoma mansoni* experimentally infected rodents and primates. A meta-analytical approach. Int J Parasitol..

[23] Williams CL, Gilbertson DE (1983). Altered feeding response as a cause for the altered heartbeat rate and locomotor activity of *Schistosoma mansoni*-infected *Biomphalaria glabrata*. J Parasitol..

[24] Campbell SJ, Stothard JR, O’Halloran F, Sankey D, Durant T, Ombede DE (2017). Urogenital schistosomiasis and soil-transmitted helminthiasis (STH) in Cameroon: an epidemiological update at Barombi Mbo and Barombi Kotto crater lakes assessing prospects for intensified control interventions. Infect Dis Pov.

[25] Trewasas P, Green J, Corbet SA (1972). Ecological studies on crater lakes in West Cameroon Fishes of Barombi Mbo. J Zool..

[26] Muir AP, Biek R, Thomas R, Mable BK (2014). Local adaptation with high gene flow: temperature parameters drive adaptation to altitude in the common frog (*Rana temporaria*). Mol Ecol..

[27] O’Donnell DR, Hamman CR, Johnson EC, Kremer CT, Klausmeier CA, Litchman E (2018). Rapid thermal adaptation in a marine diatom reveals constraints and trade-offs. Glob Chang Biol..

[28] Card DC, Schield DR, Castoe TA (2018). Plasticity and local adaptation explain lizard cold tolerance. Mol Ecol..

[29] Rey O, Estoup A, Vonshak M, Loiseau A, Blanchet S, Calcaterra L (2012). Where do adaptive shifts occur during invasion? A multidisciplinary approach to unravelling cold adaptation in a tropical ant species invading the Mediterranean area. Ecol Lett..

[30] Sternberg ED, Thomas MB (2014). Local adaptation to temperature and the implications for vector-borne diseases. Trends Parasitol..

[31] Johansson MP, Laurila A (2017). Maximum thermal tolerance trades off with chronic tolerance of high temperature in contrasting thermal populations of *Radix balthica*. Ecol Evol..

[32] Kalinda C, Chimbari M, Mukaratirwa S (2017). Implications of changing temperatures on the growth, fecundity and survival of intermediate host snails of schistosomiasis: a systematic review. Int J Environ Res Public Health..

[33] El-Hassan AA (1974). Laboratory studies on the direct effect of temperature on *Bulinus truncatus* and *Biomphalaria alexandrina*, the snail intermediate hosts of schistosomes in Egypt. Folia Parasitol (Praha)..

[34] Kalinda C, Chimbari MJ, Mukaratirwa S (2017). Effect of temperature on the *Bulinus globosus*-*Schistosoma haematobium* system. Infect Dis Poverty..

[35] Marti H (1986). Field observations on the population dynamics of *Bulinus globosus*, the intermediate host of *Schistosoma haematobium* in the Ifakara area, Tanzania. J Parasitol..

[36] Martínez-Ortí A, Bargues MD, Mas-Coma S (2015). Dos nuevas localizaciones para España de *Bulinus truncatus* (Audouin, 1827) (Gastropoda, Planorbidae), hospedador intermediario de schistosomiasis urinaria. AMZ..

[37] Welter-Schultes FW (2012). European non-marine molluscs, a guide for species identification.

[38] Majoros G, Feher Z, Deli T, Foldvari G (2008). Establishment of *Biomphalaria tenagophila* snails in Europe. Emerg Infect Dis..

[39] McCreesh N, Booth M (2014). The effect of simulating different intermediate host snail species on the link between water temperature and schistosomiasis risk. PLoS One..

[40] Coelho JR, Bezerra FS (2006). The effects of temperature change on the infection rate of *Biomphalaria glabrata* with *Schistosoma mansoni*. Mem Inst Oswaldo Cruz..

[41] Roushdy MZ, el-Emam M (1984). The effect of temperature on inter-relationship between *Schistosoma haematobium* and *Bulinus truncatus* in Egypt. J Egypt Soc Parasitol..

[42] Pfluger W, Roushdy MZ, El Emam M (1984). The prepatent period and cercarial production of *Schistosoma haematobium* in *Bulinus truncatus* (Egyptian field strains) at different constant temperatures. Z Parasitenkd..

[43] Chu KY, Massoud J, Sabbaghian H (1966). Host-parasite relationship of *Bulinus truncatus* and *Schistosoma haematobium* in Iran. 3. Effect of water temperature on the ability of miracidia to infect snails. Bull World Health Organ..

[44] Pfluger W (1980). Experimental epidemiology of schistosomiasis. I. The prepatent period and cercarial production of *Schistosoma mansoni* in *Biomphalaria* snails at various constant temperatures. Z Parasitenkd..

[45] Basch PF (1991). Schistosomes: development, reproduction, and host relations.

[46] Rubaba O, Chimbari MJ, Mukaratirwa S (2016). The role of snail aestivation in transmission of schistosomiasis in changing climatic conditions. Afr J Aquat Sci..

[47] Woolhouse MEJ, Taylor P (1990). Survival rates of *Bulinus globosus* during aestivation. Ann Trop Med Parasitol..

[48] White MM, Fried B, Sherma J (2007). Effects of aestivation and starvation on the neutral lipid and phospholipid content of *Biomphalaria glabrata* infected with *Schistosoma mansoni*. J Parasitol..

[49] Barbosa FS, Barbosa I (1958). Dormancy during the larval stages of the trematode *Schistosoma Mansoni* in snails estivating on the soil of dry natural habitats. Ecology..

[50] Badger LI, Oyerinde JPO (1996). *Schistosoma mansoni*: effect of aestivation on the intra-molluscan stages and the survival rate of infected *Biomphalaria pfeifferi*. Ann Trop Med Parasitol..

[51] Houghton JT, Ding Y, Griggs DJ, Noguer M, van der Linden PJ, Dai X, et al. Climate change 2001: the scientific basis. In: Contribution of Working Group I to the third assessment report of the intergovernmental panel on climate change. Cambridge, UK and New York, USA: CUP; 2001.

[52] Cordellier M, Pfenninger M (2009). Inferring the past to predict the future: climate modelling predictions and phylogeography for the freshwater gastropod *Radix balthica* (Pulmonata, Basommatophora). Mol Ecol..

[53] Pedersen UB, Stendel M, Midzi N, Mduluza T, Soko W, Stensgaard AS (2014). Modelling climate change impact on the spatial distribution of fresh water snails hosting trematodes in Zimbabwe. Parasit Vectors..

[54] Tanser F, Azongo DK, Vandormael A, Barnighausen T, Appleton C (2018). Impact of the scale-up of piped water on urogenital schistosomiasis infection in rural South Africa. Elife..

